# P13 DMARD preconception counselling and reinforcement should be a norm in all rheumatology clinics

**DOI:** 10.1093/rap/rkae117.044

**Published:** 2024-11-05

**Authors:** Kapil Garg, Anupama Nandagudi

**Affiliations:** Basildon University Hospital, Mid and South Essex NHS Foundation Trust, Basildon, United Kingdom; Basildon University Hospital, Mid and South Essex NHS Foundation Trust, Basildon, United Kingdom

## Abstract

**Introduction:**

Leflunomide is known to be teratogenic in animal studies, particularly in rats, with increased risk of malformations and reduced foetal viability. The FDA labelled leflunomide as a class X drug and the BSR 2022 guidelines recommend discontinuation of leflunomide at least two years before attempting to conceive. Human data supporting these recommendations are sparse. On the contrary, there are many small-medium sized case series which report no demonstrable harm caused by leflunomide to foetus or mother. We present a case of young female with rheumatoid arthritis (RA) on leflunomide who became pregnant and discovered at nine weeks of gestation.

**Case description:**

We present case of 36-year-old female who had three pregnancies previously in 2006, 2011 and 2013 and delivered healthy babies without any complications. She was not on any medication at that time. She was diagnosed with seropositive RA in 2016 and was managed on methotrexate for the first two years but was stopped due to hair loss. She was started on hydroxychloroquine and sulfasalazine with partial response. She was commenced on leflunomide in 2018 after counselling on need for contraception and teratogenic effects of leflunomide. She had to come off adalimumab in 2021 after brief use due to weight gain. She also had issues with adherence to medications as well as blood monitoring over the years. She was started on rituximab infusion in June 2022 in view of high DAS-28 score of 6.57 and subsequent received IM depomedrone injection in August 2022.

She was reviewed in a routine rheumatology clinic in November 2022 when she informed us that she was 15 weeks pregnant. Her pregnancy was diagnosed at nine weeks gestation; her latest obstetric ultrasound in October 2022 estimated gestation age at 10 weeks and four days. She was asked to stop leflunomide and informed of risks involved. We addressed her concerns about her pregnancy and liaised with the obstetric team about close monitoring of baby. We contacted UKTIS urgently. The advice was that there is not much evidence of teratogenicity in humans; most of the data are from animal studies and thus there is not much evidence of benefit with colestyramine where baby is already past the organ formation stage. The patient was promptly counselled about teratogenicity risk and advice received from UKTIS. She was not keen on colestyramine, being extremely nauseous, and was commenced on folic acid.

**Discussion:**

We later discovered that patient had her contraceptive implant removed earlier in 2022 due to menorrhagia. Regularly re-enforcing the need for contraception in reproductive age female patients on at risk DMARDs might help prevent these events. As her DAS-28 was high, she was managed with IM depomedrone, HCQ and SSZ during rest of her pregnancy. The patient was closely followed in rheumatology clinic to manage her RA disease activity. She underwent regular obstetric check-ups and scans which were reported normal. She delivered a healthy male baby in May 2023 with induction. Her baby is currently 13 months old and doing very well. Her leflunomide was restarted in September 2023 with detailed advice on need for strict contraception while using leflunomide, which she seems to be following currently.

The advice from UKTIS was useful and reassuring. This helped us promptly counsel patient comprehensively and address her concerns effectively. Although the manufacturer recommends an 11-day washout using colestyramine or activated charcoal, this was not recommended by UKTIS in this case, as the patient was 14+ weeks gestation. We did contemplate sending leflunomide levels but were discouraged by long turnaround time of drug levels, which is practically not very helpful. The decision should therefore rest on clinical assessment and discussion about risks and benefits of situation. The option of washout also should be offered to the patient as all options should be discussed in detail, which we did. Our patient did not choose to go for the washout procedure and was happy to carry on with pregnancy with close monitoring by the obstetrics department.

**Key learning points:**

• Leflunomide administration should be interrupted at least two years before a planned pregnancy and contraception must be used during this period.

• In the case of unplanned pregnancy, the use of leflunomide should be stopped as soon as possible and further advice sought from UKTIS including washout procedure.

• Close monitoring and management in a multi-disciplinary approach with the help of obstetric colleagues should be done once drug exposure is confirmed

• Drug levels are recommended by the manufacturer but are not very practical and helpful in making immediate decision due to long turnaround time.

• We should reinforce the advice on contraception on each visit in reproductive age group patients who are on drugs with likely risk of teratogenicity. This should be treated as never-events and easily avoidable.

• Although there are no convincing human data to suggest teratogenicity, we should err towards caution until more data are available. This needs further evaluation.

